# Intravenous methadone for pain management in cardiac surgery: a randomised controlled trial with plasma concentration analysis*

**DOI:** 10.1111/anae.16754

**Published:** 2025-08-26

**Authors:** Henry Man Kin Wong, Veronica Ka Wai Lai, Sandra Lok Ching Chiu, Wai Tat Wong, Siu Kwan Wo, Joan Zhong Zuo, Xiaodong Liu, Randolph Hung Leung Wong, Kwok Ming Ho

**Affiliations:** ^1^ Department of Anaesthesia and Intensive Care The Chinese University of Hong Kong Hong Kong SAR China; ^2^ Child Health Evaluative Sciences, The Hospital for Sick Children Research Institute Toronto Ontario Canada; ^3^ School of Pharmacy, Faculty of Medicine, The Chinese University of Hong Kong Hong Kong SAR China; ^4^ Division of Cardiothoracic Surgery, Department of Surgery, Prince of Wales Hospital, New Territories Hong Kong SAR China

**Keywords:** cardiac surgical procedures, intravenous methadone, methadone plasma concentration analysis, postoperative pain management, sternotomy pain management

## Abstract

**Introduction:**

Postoperative pain after cardiac surgery remains significant despite the administration of opioids. Methadone may improve pain control and decrease the need for postoperative opioids. Randomised controlled trials, however, are limited and the effects of cardiopulmonary bypass on methadone pharmacokinetics are unclear. The aims of this study were to compare methadone and morphine in cardiac surgery, measuring methadone concentrations and correlating them with pain control.

**Methods:**

Patients undergoing cardiac surgery that required cardiopulmonary bypass were allocated randomly to receive either 0.2 mg.kg^‐1^ methadone or 0.2 mg.kg^‐1^ morphine (based on actual body weight, maximum 20 mg for both drugs). Postoperative pain was assessed at 15 min and 8 h, 12 h, 24 h, 48 h and 72 h after tracheal extubation, by analysis of morphine consumption and pain scores. Opioid‐related adverse events were evaluated. Postoperative blood samples were collected for 96 h to measure plasma methadone concentrations.

**Results:**

In total, 80 patients were analysed (40 allocated to the methadone group, 40 allocated to the morphine group). Patients allocated to the methadone group had significantly reduced 24‐h and total postoperative morphine requirements compared to those allocated to the morphine group (median (IQR [range]) 9 (5–16 [0–40]) mg vs. 24 (17–43 [4–54]) mg (p < 0.001) at 24 h and 35 (23–52 [5–66]) mg vs. 11 (7–20 [0–44]) mg (p < 0.001) total). Patients allocated to the methadone group had lower pain scores at rest (β ‐2.24, standard error 0.49, p < 0.001) and on coughing (β ‐2.16, standard error 0.50, p < 0.001). There was no difference in the incidence of opioid‐related adverse effects between the two groups. Plasma methadone concentration decreased during cardiopulmonary bypass but remained above the minimum effective analgesic concentration for approximately 24 h after administration (mean (SD) 51 (24.7) ng.ml^‐1^ at baseline to 30 (10.7) ng.ml^‐1^ at 24 h).

**Discussion:**

Intra‐operative methadone reduces postoperative analgesia requirements without increasing the incidence of opioid‐related adverse events.

## Plain Language Summary

After heart surgery, many people still feel a lot of pain, even when they get strong pain medicine like opioids. Methadone is another medicine that might help control pain better and reduce the need for more opioids, but scientists have not done many careful studies on this, and they are not sure how the heart–lung machine (used during surgery) affects methadone in the body. This study looked at how well methadone works compared to morphine during heart surgery and also measured how much methadone stayed in the blood. People who were having heart surgery with a heart–lung machine were split into two groups. One group got methadone and the other got morphine. Each person got the same amount based on their weight. After surgery, their pain was checked at several times: 15 minutes, 8 hours, 12 hours, 24 hours, 48 hours and 72 hours after they were taken off the breathing machine. The doctors looked at how much extra morphine the patients needed and how much pain they felt. They also watched for side effects. Blood samples were taken for 4 days to see how much methadone stayed in their bodies. In total, 86 people took part in the study: 44 got methadone and 42 got morphine. The methadone group needed much less morphine after surgery than the morphine group. The methadone group also said they had less pain, both when resting and when coughing. Both groups had about the same number of side effects. Even though methadone levels dropped during the surgery, the amount in the blood stayed high enough to help with pain for about a day. Giving methadone during heart surgery helped reduce pain afterwards and meant patients needed less extra pain medicine. It did not cause more side effects.

## Introduction

Postoperative pain remains significant after cardiac surgery [1, 2]. Poorly controlled pain contributes to adverse outcomes including: delayed ambulation; decreased patient satisfaction; chronic postsurgical pain; and cardiopulmonary complications [3, 4, 5]. Current opioid‐based pain management strategies in cardiac surgery may lead to fluctuating blood concentrations, inadequate analgesia or respiratory suppression [6]. Repeated opioid administration also increases the risk of tolerance and opioid‐induced hyperalgesia [7], further complicating pain management and potentially leading to chronic opioid use [8]. It is therefore imperative to have a long‐acting drug that decreases opioid consumption while managing postoperative pain.

Methadone, a long‐acting opioid, offers potential advantages. It has a prolonged duration of action [9, 10] and mitigates tolerance and hyperalgesia through N‐methyl‐D‐aspartate receptor and neurotransmitter modulation [7, 11, 12]. While some studies suggest benefits with intra‐operative methadone [13, 14, 15, 16, 17], there are only a limited number of randomised controlled trials investigating the use of methadone in cardiac surgery. Specifically, there is a lack of robust randomised controlled trials comparing methadone with morphine [16, 17, 18], which is the standard opioid analgesia used in many cardiac surgical centres.

Despite recent data on the impact of cardiopulmonary bypass (CPB) on methadone pharmacokinetics [19, 20, 21], the relationship between methadone plasma concentrations and postoperative pain control in cardiac surgery has not been investigated thoroughly. Given the additional non‐opioid receptor‐related anti‐hyperanalgesic and anti‐allodynic properties, the prolonged clinical response of methadone could be multifaceted, beyond its therapeutic plasma level, which could be shown with plasma concentration analysis.

We hypothesised that, compared with morphine, intra‐operative methadone use in adult patients having cardiac surgery would provide superior analgesia and reduce postoperative morphine requirements without increasing adverse effects. To determine whether methadone is superior to morphine, we conducted a randomised controlled trial to compare the efficacy and safety of methadone with morphine. In addition, we characterised the relationship between methadone plasma concentrations and its analgesia effects up to 96 h after administration.

## Methods

This was a single‐centre, double‐blinded, randomised controlled trial. After institutional and ethical approval, a pilot study was initially conducted to evaluate the safety and analgesic efficacy of methadone in cardiac surgery, a novel application in Hong Kong. Upon confirming the safety and feasibility of methadone for peri‐operative pain management, evidenced by the absence of unexpected arrhythmias and timely tracheal extubation, a randomised controlled trial was initiated based on the initial findings. While chronic postsurgical pain data, including Douleur Neuropathique 4 (DN4) and Brief Pain Inventory (BPI), were collected as per the trial registry, here we focus on acute pain outcomes. The chronic pain data will inform future research exploring the role of methadone in chronic postsurgical pain following cardiac surgery.

Adult patients aged ≥ 18 y, undergoing elective coronary artery bypass graft (CABG), valve repair/replacement or combined CABG/valve procedure via sternotomy, were eligible for inclusion. Written informed consent was obtained from all patients. Patients were not studied if they met any of the following criteria: emergency, aortic or redo surgery; pre‐operative renal failure requiring renal replacement therapy or creatinine clearance < 30 ml.min^‐1^ (calculated by Cockcroft–Gault formula); liver dysfunction (liver enzymes twice upper limit of normal); left ventricular ejection fraction < 40%; requiring mechanical haemodynamic support in the pre‐operative period; history of chronic pain or regularly used pain medications; history of psychiatric illnesses or illicit drug use; intra‐operative use of remifentanil; or unable to provide informed consent.

Patients were assigned randomly to receive either 0.2 mg.kg^‐1^ methadone or 0.2 mg.kg^‐1^ morphine (dose based on actual body weight up to a maximum of 20 mg for both drugs, added to a syringe containing saline made up to 50 ml in total) by drawing sequentially numbered, coded sealed, opaque envelopes each containing the assignment. The sealed envelopes for randomisation were prepared by a third party who took no further part in the study. Random allocation was performed in a 1:1 ratio using RStudio (R Foundation, Vienna, Austria) in permuted blocks of size four. The study syringes containing the drug solution were prepared with blinded labelling by a nurse or anaesthetist not involved in the study. The surgical team, blinded to the group allocation, performed standardised surgical procedures. Blinded anaesthetists and nurses collected data intra‐operatively, in the ICU and wards.

All patients had standard cardiac surgery monitoring applied. General anaesthesia was induced with midazolam 0.01–0.05 mg.kg^‐1^, fentanyl 2–5 μg.kg^‐1^ and rocuronium 0.5–1 mg.kg^‐1^ to facilitate tracheal intubation. Anaesthesia was maintained with sevoflurane and a propofol infusion, targeting a bispectral index of 40–60. The study drug was administered intravenously over 30 min at induction.

Methadone–morphine dose conversion is limited and often based on institutional experience [22]. Most literature used comparative dosing [16, 17, 18, 23] and 0.2 mg.kg^‐1^ is a typical intra‐operative morphine dose in our centre [24]. No further opioids, analgesics (including regional techniques), steroids or anti‐emetics were administered intra‐operatively. Hypertension was managed by increasing the rate of the propofol infusion or concentration of sevoflurane, or by nitroglycerine boluses if the bispectral index dropped below the target.

After ICU admission, the propofol infusion was stopped to facilitate weaning from the ventilator using adaptive support ventilation, which adjusts the ventilation parameters depending on the lung mechanics and effort of the patient. After tracheal extubation, patients were assessed for pain score and level of sedation at 15 min and 8 h, 12 h, 24 h, 48 h and 72 h. The postoperative analgesia protocol was identical in both study groups, including patient‐controlled analgesia programmed to administer 1 mg morphine with a lockout interval of 6 min for 72 h after surgery. Oral analgesics (paracetamol 1 g every 6 h, dihydrocodeine 30 mg three times a day) were prescribed once the patients tolerated oral intake, and on demand anti‐emetics were available (intravenous ondansetron 4 mg every 8 h). Rescue analgesics on top of the protocol regimen were prescribed as needed.

The primary outcome was postoperative morphine consumption in the 24 h after tracheal extubation. The clinical trials registry lists 72 h postoperative pain and analgesic requirements as the primary outcome, aligning with typical recovery periods. However, the most intense pain typically occurs in the first 48 h in cardiac surgery [1, 2], a critical window for recovery, leading to a maximal need for analgesia during this period, and hence, the primary outcome was adjusted. Secondary outcomes included: numerical rating scale pain scores (0–10 scale) at rest and with cough up to 72 h post‐tracheal extubation; postoperative morphine requirements up to 72 h post‐tracheal extubation; time to first morphine rescue; time to ventilation weaning; patient satisfaction up to 72 h post‐tracheal extubation (recorded on a verbal analogue scale where 0 represents worst possible experience, 100 represents best possible experience); anti‐emetics and additional analgesic requests within 72 h post‐tracheal extubation; opioid‐related adverse effects including episodes of postoperative nausea and vomiting, time to first bowel movement; and duration of ICU and hospital stay.

An exploratory analysis of plasma methadone concentrations was performed over 96 h following a single intra‐operative dose to describe the effect of CPB on methadone pharmacokinetics and determine its analgesic duration. Blood samples were collected by independent research staff blinded to the purpose of the study. The sampling protocol included 12 blood samples per patient, collected at baseline before methadone administration, at 1 h and 2 h after methadone administration to capture the initial redistribution phase and subsequently at 2‐hourly intervals until 12 h to show the decline in methadone levels. This was followed by daily sampling at 24 h, 48 h, 72 h and 96 h after administration to determine the drug clearance. A one‐sample t‐test was conducted at each time point to assess whether the mean plasma methadone concentration exceeded the minimum effective analgesic concentration (MEAC) of 30 ng.ml^‐1^ [25], with statistical significance defined as p < 0.05.

The sample size was calculated using G*Power 3.1.9.3 (Kiel University, Kiel, Germany) based on postoperative morphine consumption 24 h post‐tracheal extubation, which was the primary outcome variable. Without a priori data on methadone use for cardiac surgery in the Chinese population, a pilot study with 50 patients (unpublished) was conducted in Prince of Wales Hospital, Hong Kong. The mean (SD) postoperative morphine consumption at 24 h was 14.4 (8.4) mg in patients receiving methadone, whereas for the morphine group, it was 23.6 (14.2) mg. With an α probability of 0.05 and a power of 90%, a sample size of 34 patients in each group was needed. Assuming a 20% drop‐out rate, the sample size was increased to 86 patients. The outcomes were analysed and reported on a per‐protocol basis. Since the pain scores collected over time are correlated, generalised estimated equation models were used to analyse the postoperative analgesia time effects. Comparison of continuous data was performed by independent sample t‐tests and Mann–Whitney U‐test for parametric and nonparametric variables respectively. Categorical variables were compared using χ^2^ tests. Data analyses were carried out using SPSS 27.0 (IBM Corp, Armonk, NY) and Stata V.14 (Stata, College Station, TX, USA) was used to conduct a generalised estimated equation model with a Gaussian distribution, identity‐link function, exchangeable correlation with robust standard error. The level of significance was set at p < 0.05 without adjusting for multiple testing.

## Results

**Figure 1:**
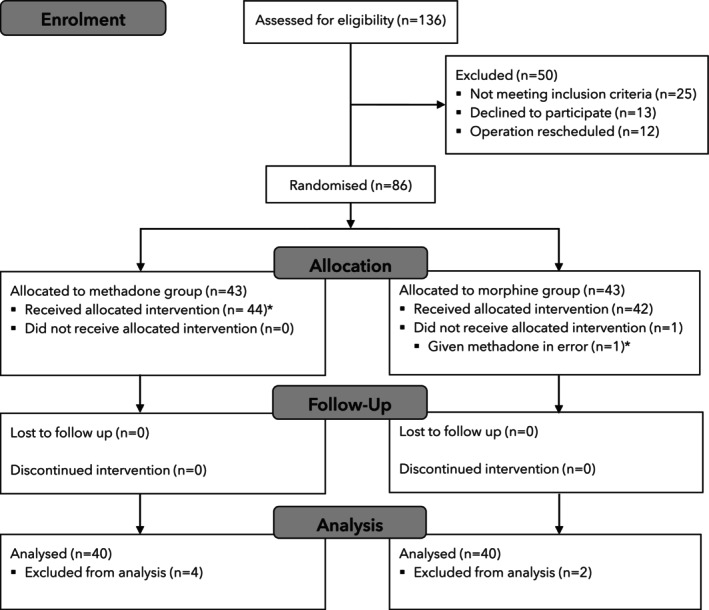
Study flow diagram. *One patient was given methadone instead of morphine due to misreading of the group assignment, which was found during plasma concentration analysis. This patient was analysed per protocol in the methadone group.

Of the 136 patients assessed between January 2023 and November 2024, 50 were not included (Fig. 1) and 86 patients were allocated randomly 1:1 to the methadone or morphine groups. One patient allocated to the morphine group was administered inadvertently with methadone, which was noticed during plasma concentration analysis and was reassigned. Final analysis included 44 patients in the methadone group and 42 patients in the morphine group. Baseline characteristics were comparable (Table 1). Four patients allocated to the methadone group were not included in the outcome analysis due to surgical complications (re‐sternotomy for haemostasis, cardiogenic shock, airway injury from transoesophageal echocardiography and delirium). Two patients allocated to the morphine group were not included in the analysis due to prolonged weaning from severe bronchospasm and complicated bleeding.

**Table 1:** Characteristics of enrolled patients. Values are mean (SD), number or median (IQR [range]).

	Methadone group	Morphine group
	n = 44	n = 42
Age; y	62 (10.7)	65 (8.7)
Sex; male	33	30
Weight; kg	66 (11.7)	68 (13.2)
BMI; kg.m^‐2^	24.4 (3.8)	25.1 (4.6)
NYHA classification		
1	9	10
2	27	25
3	6	7
4	2	0
Logistic EuroScore	1.8 (1.4–4.2 [0.9–11.1])	2.3 (1.5–3.1 [0.9–9.9])
Baseline LVEF; %	58 (7.7)	59 (6)
Type of cardiac surgery		
CABG	18	16
Single valve	24	25
Multiple valves	1	0
CABG plus valves	1	0
Others	0	1
Fentanyl at induction; μg	250 (250–313 [150–450])	250 (250–350 [100–450])
Midazolam at induction; mg	1.8 (0–2.0 [0–5.0])	2.0 (1.0–3.0 [0–3.0])
Surgery time; min	258 (59.0)	242 (53.1)
CPB time; min	120 (100–149 [54–236])	118 (93–143 [65–222])

NYHA, New York Heart Association; LVEF, left ventricular ejection fraction; CABG, coronary artery bypass graft; CPB, cardiopulmonary bypass.

Postoperative analgesia data are shown in Table 2. Patients allocated to the methadone group had significantly lower postoperative morphine requirements in the 24 h after tracheal extubation (median (IQR [range]) 9 (5–16 [0–40]) mg vs. 24 (17–43 [4–54]) mg, p < 0.001) and for up to 72 h (11 (7–20 [0–44]) mg vs. 35 (23–52 [5–66]) mg, p < 0.001). Time to first morphine rescue was significantly longer in patients allocated to the methadone group (median (IQR [range]) 205 (103–397 [52–4485]) min vs. 134 (82–214 [8–290]) min, p = 0.041). Patients allocated to the methadone group used less morphine at all postoperative 12‐h intervals (Table 2), with two patients requiring none. The overall satisfaction with pain management was better among patients allocated to the methadone group at all time points (Table 2).

**Table 2:** Postoperative clinical outcomes, postoperative morphine requirements, pain score at rest and on coughing from 15 min to 72 h after tracheal extubation, and overall satisfaction with pain management. Values are median (IQR [range]). Six enrolled patients were excluded in the analyses due to postoperative complications.

	Methadone group	Morphine group	p value
	n = 40	n = 40	
Time of ventilation weaning to spontaneous breathing; min	240 (151–365 [87–780])	223 (176–325 [100–645])	0.868
Time to tracheal extubation; min	330 (242–535 [165–1055])	306 (280–416 [160–691])	0.533
Duration of ICU stay; h	22 (21–24 [16–116])	19 (18–23 [15–140])	0.004
Duration of hospital stay; days	8 (7–11 [5–28])	8 (6–10 [5–23])	0.302
Time to first morphine rescue; min	205 (103–397 [52–4485])	134 (82–214 [8–290])	0.041
Morphine consumption 24 h after tracheal extubation; mg	9 (5–16 [0–40])	24 (17–43 [4–54])	< 0.001
Morphine consumption after tracheal extubation; mg			
0–8 h	4 (3–9 [0–23])	14 (9–23.[2–44])	< 0.001
8–12 h	1 (0–3 [0–14])	3 (2–7 [0–13])	0.003
12–24 h	1 (1–3 [0–14])	7 (2–10 [0–24])	< 0.001
24–48 h	0 (0–2 [0–20])	5 (0–8 [0–21])	< 0.001
48–72 h	0 (0–0 [0–8])	1 (1–5 [0–15])	0.001
Total	11 (7–20 [0–44])	35 (23–52 [5–66])	< 0.001
Pain score at rest; NRS			
15 min	1 (0–2 [0–5])	3 (2–5 [0–10])	< 0.001
8 h	0 (0–2 [0–3])	1 (0–3 [0–6])	0.007
12 h	0 (0–2 [0–5])	1 (0–3 [0–6])	0.018
24 h	0 (0–1 [0–5])	1 (0–2 [0–7])	0.029
48 h	0 (0–2 [0–4])	1 (0–2 [0–6])	0.273
72 h	0 (0–1 [0–3])	0 (0–2 [0–7])	0.035
Pain score on coughing; NRS			
15 min	3 (1–5 [0–8])	5 (4–7 [1–10])	< 0.001
8 h	3 (1–4 [0–5])	4 (3–5 [0–8])	0.007
12 h	3 (1–4 [0–7])	3 (2–5 [1–8])	0.016
24 h	3 (1–4 [0–7])	3 (2–5 [0–9])	0.065
48 h	2 (1–3 [0–7])	2 (2–5 [0–8])	0.049
72 h	2 (1–4 [0–6])	3 (1–5 [0–8])	0.021
Overall satisfaction with pain management (0–100)			
12 h	80 (80–98 [50–100])	70 (53–80 [20–100])	0.003
24 h	80 (80–100 [50–100])	80 (53–80 [20–100])	< 0.001
48 h	90 (80–100 [60–100])	70 (60–80 [30–100])	< 0.001
72 h	80 (80–100 [60–100])	80 (60–80 [40–100])	< 0.001

**Figure 2:**
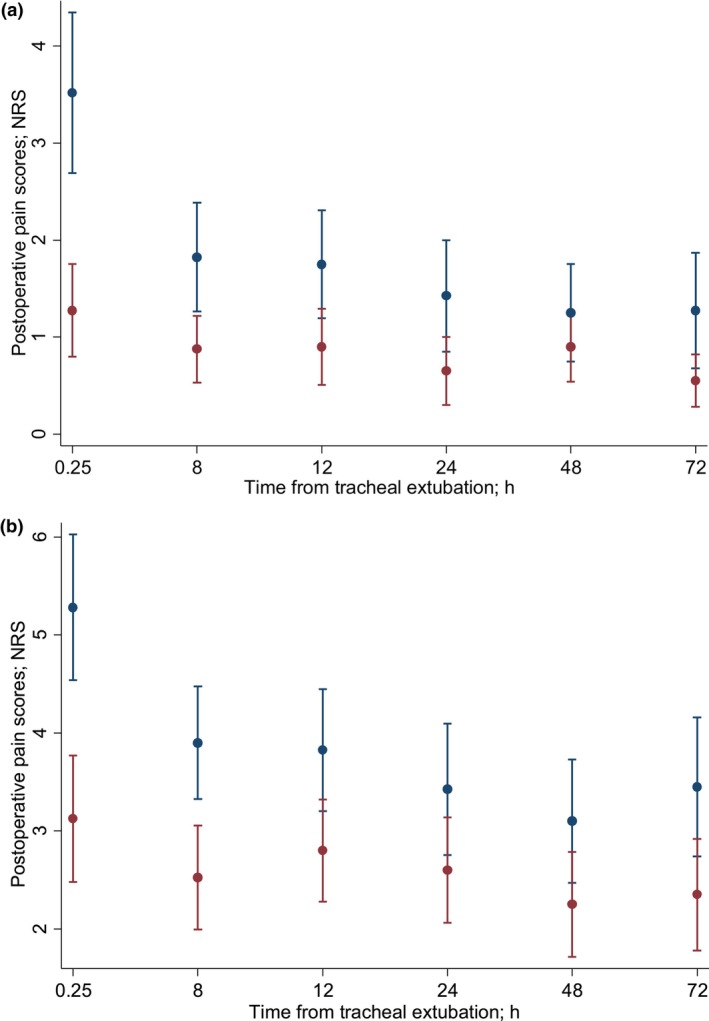
Generalised estimated equation analysis on postoperative pain scores at rest (a) and during coughing (b), in methadone (red) and morphine (blue) group, up to 72 h after tracheal extubation. Values are mean (95%CI). NRS, numerical rating scale (0–10).

Generalised estimating equation models were conducted to evaluate the marginal analgesia time effects between groups, pain scores at rest and coughing and their interactions (Fig. 2). Patients allocated to the methadone group exhibited significantly lower pain scores (β ‐2.24, standard error 0.49, p < 0.001). A significant interaction was observed between patients allocated to the methadone group and timing (χ^2^ [5] = 17.87, p = 0.003), suggesting that the treatment effect was amplified with time. Predictive margins further illustrated these effects, with mean (SD) numerical rating scale scores in patients allocated to the methadone group decreasing from 1.28 (1.55) at 15 min post‐tracheal extubation to 0.55 (0.88) at 72 h post‐tracheal extubation, compared with a decrease from 3.52 (2.67) at 15 min to 1.27 (1.94) at 72 h in those allocated to the morphine group. Patients allocated to the methadone group displayed significantly lower pain scores (β ‐2.16, standard error 0.50, p < 0.001) on coughing. Taken together, the results from the generalised estimated equation models reveal that methadone was associated with consistently lower postoperative pain scores, both at rest and during coughing, across the study population over time.

No differences in opioid‐related adverse effects were observed within 72 h of surgery (online Supporting Information Table S1), including nausea and vomiting; requirement for rescue anti‐emetics; and the number of anti‐emetic requests. Time to first bowel movement and Ramsay Sedation Scores were similar between groups at all measurement time‐points. There were no differences between groups in time of ventilator weaning to spontaneous breathing; time to tracheal extubation; and duration of hospital stay (Table 2). Duration of ICU stay was significantly longer in patients allocated to the methadone group but only by 3 h (median (IQR [range]) 22 (21–24 [16–116]) h vs. 19 (18–23 [15–140]) h, p = 0.004).

**Figure 3:**
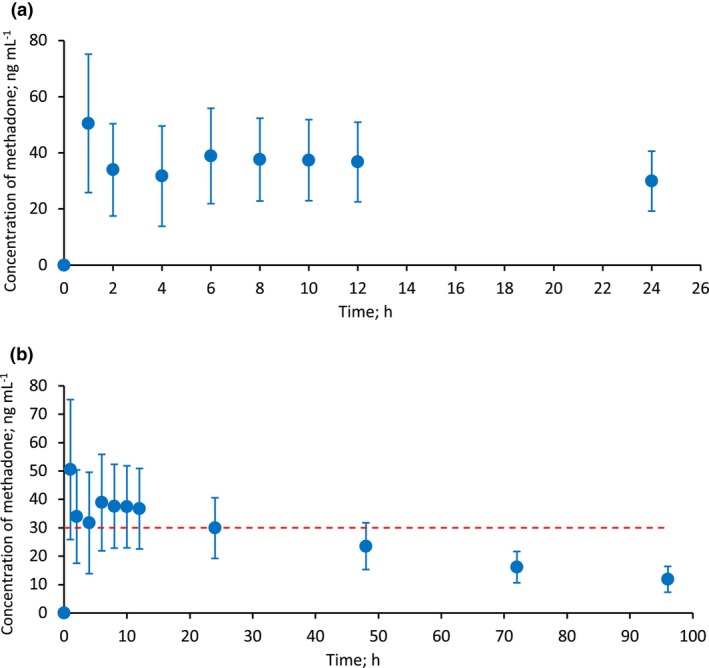
Plasma methadone concentration at sampling times during the study period: (a) from the time of methadone administration until 24 h after the single bolus dose; (b) from the time of methadone administration until 96 h after the single bolus dose. The red dotted line represents the minimum effective analgesic concentration at 30 ng.ml^‐1^ determined by Gourlay et al. [25]. Values are mean (SD) of plasma methadone concentration at each sampling time.

Figure 3 summarises the changes in plasma methadone concentration. A significant drop in plasma level was observed after CPB initiation (mean of 78 min after methadone administration and discontinued at a mean of 213 min). Samples for methadone concentration were taken at a mean time of 33 min before CPB initiation and at a mean time of 19 min after CPB ended (mean (SD) concentrations 51 (24.7) ng.ml^‐1^ and 32 (17.9) ng.ml^‐1^, respectively); these indicated a 37% decrease in plasma methadone concentration. A slight increase in methadone concentration was observed after CPB cessation (Fig. 3).

Table 3 shows a series of one‐sample t‐tests to evaluate whether the mean plasma concentration of methadone at various time points differed significantly from the MEAC of 30 ng.ml^‐1^ [25]. Mean (SD) plasma concentrations were significantly higher than 30 ng.ml^‐1^ at 1 h, 8 h and 12 h (Table 3). In contrast, the plasma concentration at 48 h, 72 h and 96 h was significantly lower than 30 ng.ml^‐1^ (Table 3). This establishes that the mean plasma methadone concentration declined over time, falling below the MEAC around 24 h post‐administration (Fig. 3 and Table 3). Despite this, analgesia persisted for 48–72 h post‐tracheal extubation (corresponding to 60–84 h after methadone administration, respectively), evidenced by a significant interaction with pain scores, better satisfaction and reduced morphine requirements.

**Table 3:** Comparison between plasma concentration of methadone (n = 44) at various time points from the minimum effective analgesic concentration at 30 ng.ml^‐1^ as determined by Gourley et al. [25]. Values are mean (SD) with 95 % CI.

Time from methadone administration	Time after tracheal extubation	Plasma concentration of methadone (ng.ml^‐1^)	p‐value	Mean difference	95%CI
1 h*		51 (24.7)	< 0.001	20.5	13.0–28.0
2 h		34 (16.4)	0.117	4.0	‐1.0–9.0
4 h		32 (17.9)	0.526	1.7	‐3.7–7.2
6 h*		39 (17.0)	0.001	8.9	3.7–14.1
8 h*		38 (14.8)	0.001	7.6	3.1–12.1
10 h*		37 (14.4)	0.001	7.4	3.0–11.8
12 h*	15 min	37 (14.1)	0.003	6.7	2.4–11.1
24 h	12 h	30 (10.7)	0.961	‐0.1	‐3.3–3.2
48 h	24–48 h	24 (8.2)	< 0.001	‐6.5	9.0 to ‐4.0
72 h	48–72 h	16 (5.5)	< 0.001	‐13.9	‐15.6 to ‐12.1
96 h		12 (4.6)	< 0.001	‐18.1	‐19.6 to 16.7

* Time‐points with mean methadone concentrations exceeding the minimum effective analgesic concentration at 30 ng.ml^‐1^, as determined by a one‐sample t‐test (p < 0.05).

## Discussion

This study compared a single dose of methadone vs. a single dose of morphine for analgesia in cardiac surgery. Methadone showed superior analgesia: reduced postoperative morphine requirements by 63% at 24 h after tracheal extubation and 69% overall; improved pain scores; longer time to first rescue morphine; and increased patient satisfaction. Methadone provided better analgesic effects at rest and at 12 h, 24 h and 48 h post‐tracheal extubation on coughing. Opioid‐related adverse effects were similar between groups. Collectively, the study suggests that intra‐operative methadone is effective and safe for postoperative analgesia in cardiac surgery. A slightly longer ICU duration of stay in the methadone group likely reflected logistical factors such as bed availability.

The use of a lower dose of methadone (0.2 mg.kg^‐1^) has not been well studied in cardiac surgery. A systematic review suggested that, at a dose of 0.1–0.3 mg.kg^‐1^, methadone reduces opioid use and pain scores without increasing opioid‐related adverse events, which was compatible with our findings [16]. However, heterogeneity in study design and small sample sizes hinder definitive conclusions. A study using 0.3 mg.kg^‐1^ methadone also showed benefits [6]; however, a recent retrospective dose–response observational study linked higher doses (> 0.3 mg.kg^‐1^) to more adverse effects including postoperative nausea and vomiting; delirium; and increased duration of hospital stay. Notably, low‐dose (< 0.25 mg.kg^‐1^) and medium‐dose (0.25–0.3 mg.kg^‐1^) methadone have shown promise for pain reduction and opioid sparing. Carvalho et al. allocated patients having cardiac surgery to receive either 0.1 mg.kg^‐1^ methadone or morphine at the end of surgery [17]. Despite lower pain scores in the patients allocated to the methadone group 24 h after surgery, they found no difference in pain scores at 12 h and 36 h after surgery. A retrospective cohort study in patients having CABG and receiving 0.2 mg.kg^‐1^ methadone as part of multimodal analgesia showed benefits until postoperative day 2 [26]. The ideal dose of methadone requires a balance of efficacy and adverse effects, aligning with guidelines on pain management and opioid stewardship in cardiac surgery [27].

This is one of the few randomised controlled trials comparing a single administration of methadone (0.2 mg.kg^‐1^) with morphine at anaesthesia induction in patients having cardiac surgery, indicating analgesia efficacy of intra‐operative methadone at this dose. Given potential haemodilution from CPB, documenting methadone concentrations relative to its previously documented MEAC (approximately 30–60 ng.ml^‐1^) [25, 28] is crucial to understanding its analgesic effect.

Methadone is metabolised primarily by cytochrome P450 (CYP) 2B6, which has similar hepatic abundance and phenotype frequencies between Caucasians and Chinese [29]; we therefore adopted the established MEAC. Methadone levels remained above the MEAC for approximately 24 h (Fig. 3 and Table 3), correlating with reduced pain scores at 12 h post‐tracheal extubation (Table 2). Analgesia persisted up to 72 h post‐tracheal extubation (> 80 h post‐administration), shown by reduced postoperative morphine requests and better patient satisfaction (Table 2), possibly attributed to the non‐opioid mechanisms of methadone, such as modulating neurotransmitters reuptake and antagonising N‐methyl‐D‐aspartate receptors, which mitigates opioid tolerance and hyperalgesia [30, 31].

This study shows the efficacy of lower dose intra‐operative methadone in cardiac surgery, supporting opioid stewardship [26]. It provides the first evidence of extended analgesia beyond the MEAC, potentially reducing long‐term opioid use [8]. The peri‐operative analgesia protocol was well controlled, minimising the confounding drug effects on postoperative analgesia outcomes. Limitations include the single‐centre design and methadone concentration sampling only in patients allocated to the methadone group, which might introduce bias despite blinding and independent blood sampling. In addition, due to the non‐opioid receptor activities of methadone, the equivalent analgesic doses of methadone and morphine have not been established. Conversion ratios ranging from 1:1 to 1:14 have been reported [32]. Mathematical models suggested a nonlinear, parabolic relationship [33], with lower conversion ratios at lower morphine doses and higher conversion ratios at higher morphine doses, especially in patients who require more than 100 mg of morphine per day. According to the Ayonrinde equation (methadone (mg) = √[2.3 × morphine (mg)] + 15), at low doses, oral methadone (0.2 mg.kg^‐1^) is not dissimilar to morphine [33, 34]. Oral‐to‐parenteral methadone conversion has been reported from 1:0.7 to 1:2 [35], supporting the 1:1 parenteral methadone‐to‐morphine ratio used in this study. From the limited information in the literature, 0.2 mg.kg^‐1^ methadone could have a stronger effect on the opioid receptors than 0.2 mg.kg^‐1^ morphine [22, 36]. Nonetheless, our results showed that the morphine requirement at 24 h post‐tracheal extubation in the methadone group was only about one‐third of that in the morphine group, suggesting that differences in their potency on the opioid receptors were unlikely to account for the superior analgesic effects of methadone we observed in this study. In addition, the MEAC was established in a Caucasian cohort undergoing general abdominal and spinal surgery, but the similar prevalence of hepatic CYP2B6, the primary enzyme metabolising methadone, could support the application of a similar MEAC in Chinese populations.

In conclusion, a single dose of methadone (0.2 mg.kg^‐1^) at induction of anaesthesia significantly reduced postoperative pain and opioid needs, and improved patient satisfaction up to 72 h after surgery, including the period when methadone plasma levels were below the MEAC. Methadone shows promise as part of the multimodal analgesic plan in enhancing recovery after adult cardiac surgery. Future research should explore its impact on chronic postsurgical pain in cardiac surgery.

## Supporting information


**Table S1.** Postoperative opioid‐related complications, use of rescue antiemetics and analgesics within 72 h of surgery.
