# Supplementary material for: Intravenous methadone for pain management in cardiac surgery: a randomised controlled trial with plasma concentration analysis*
Source: Anaesthesia. 2025 Aug 26;81(1):51–61. doi: 10.1111/anae.16754 (PMC12747610; doi:10.1111/anae.16754)
Supplement: Supplementary file 1 — Table S1. Postoperative opioid‐related complications, use of rescue antiemetics and analgesics within 72 h of surgery. [file ANAE-81-51-s001.docx]

**Table S1** Postoperative opioid-related complications, use of rescue antiemetics and analgesics within 72 hours after surgery. Values are expressed as number (proportion) or median (IQR [range]).

|  | | **Methadone group**  **(n=40)** | **Morphine group**  **(n=40)** | **P-value** |
| --- | --- | --- | --- | --- |
| Within 72 hours after surgery | |  |  |  |
| Nausea, n (%) | 18 (45) | 18 (45) | 0.921 |  |
| Vomiting, n (%) | 12 (30) | 12 (30) | 0.943 |  |
| Number of patients required rescue antiemetic, n (%) | 31 (77.5) | 26 (65) | 0.296 |  |
| Number of antiemetic requests | 2 (0.5-4.0 [0-11]) | 1 (0-3.8 [0-8]) | 0.393 |  |
| Number of patients required rescue analgesics, n (%) | 4 (10) | 7 (17.5) | 0.331 |  |
| Time of first bowel movement, days | | 3.9 (2.3-4.9 [1.9-9.9]) | 3.6 (1.9-4.9 [1.6-9.3]) | 0.658 |
